# Ship Classification in High-Resolution SAR Images Using Deep Learning of Small Datasets

**DOI:** 10.3390/s18092929

**Published:** 2018-09-03

**Authors:** Yuanyuan Wang, Chao Wang, Hong Zhang

**Affiliations:** 1 Key Laboratory of Digital Earth Science, Institute of Remote Sensing and Digital Earth, Chinese Academy of Sciences, Beijing 100094, China; wangyy2016@radi.ac.cn; 2 College of Resources and Environment, University of Chinese Academy of Sciences, Beijing 100049, China

**Keywords:** high-resolution SAR images, ship classification, convolutional neural networks, fine tuning, transfer learning, small datasets

## Abstract

With the capability to automatically learn discriminative features, deep learning has experienced great success in natural images but has rarely been explored for ship classification in high-resolution SAR images due to the training bottleneck caused by the small datasets. In this paper, convolutional neural networks (CNNs) are applied to ship classification by using SAR images with the small datasets. First, ship chips are constructed from high-resolution SAR images and split into training and validation datasets. Second, a ship classification model is constructed based on very deep convolutional networks (VGG). Then, VGG is pretrained via ImageNet, and fine tuning is utilized to train our model. Six scenes of COSMO-SkyMed images are used to evaluate our proposed model with regard to the classification accuracy. The experimental results reveal that (1) our proposed ship classification model trained by fine tuning achieves more than 95% average classification accuracy, even with 5-cross validation; (2) compared with other models, the ship classification model based on VGG16 achieves at least 2% higher accuracies for classification. These experimental results reveal the effectiveness of our proposed method.

## 1. Introduction

Due to their all-weather, all-day, and high-resolution advantages, synthetic aperture radar (SAR) images have recently been used for ship classification in marine surveillance. There are several satellites that have provided high-resolution SAR images since 2007, such as ASI’s COSMO-SkyMed, DLR’s TerraSAR-X, Japan’s ALOS-2, and China’s Gaofen-3, These high-resolution SAR images provide a resolution greater than 3 m that contain rich information about the targets, such as the geometry of ships, which makes discriminating different types of ships possible [1,2,3,4].

The methods used for ship classification with SAR images mainly focus on feature selection and optimized classifier techniques [1,2,3,4,5,6,7,8,9,10,11,12,13]. Currently, commonly used features are (1) geometric features, such as ship length, ratio of length to width, distribution of scattering centers, covariance coefficient, contour features [11], and ship scale; and (2) scattering features, such as 2D comb features [7], local radar cross section (RCS) density [1], permanent symmetric scatterers [12], and polarimetric characteristics [13]. For classifiers, models from machine learning can be adapted, such as support vector machines [14] and artificial neural networks [15]. Besides, many researchers provide classifiers that aim for high-classification accuracy given the particularity of ships in SAR images, such as the analytical hierarchy process [2] and hierarchical scheme [3]. Since these methods are highly dependent on features and classifiers, researchers exploit several strategies to relieve the processes of feature selection and classifier optimization, such as hierarchical feature selection [6], multiple kernels to combine various features [9] and joint feature and classifier selections [8].

Since Hinton [16] integrated the single-layer, restricted Boltzmann machine into a deep neural network, deep learning, which is the automatic learning of discriminative features, has achieved enormous success in object classification, object detection, and semantic segmentation studies from normal RGB images [17]. Even in SAR images, deep-learning models are gradually used [18,19,20]. Zhu et al. [18] reviewed the potential of deep learning when applied to remote sensing and gave a list of deep-learning materials. Zhang et al. [19] surveyed perspectives for the application of deep learning to remote sensing, especially in hyperspectral communities. Nogueira et al. [20] presented three methods for six popular ConvNets on two optical datasets and one multispectral dataset and demonstrated that the method with fine-tuning features and the SVM classifier achieved the best results. Bentes et al. [21] explored convolutional neural networks for ship classification via TerraSAR-X images. The benefit of deep leaning is that unlike feature selection and optimized classifiers, it automatically learns the representation of SAR images and provides an end-to-end scheme for a given application without human interference [22], which saves time for feature extraction and selection and classifier optimization. Based on the advantages of deep learning, convolutional neural networks (CNNs) are adapted in this paper.

One of the major bottlenecks when applying deep learning is that it needs a considerably large training dataset [22]. Since this volume of labeled data is time consuming and almost impossible to obtain, there are two methods to address small datasets during training: fine tuning and transfer learning [22]. Transfer learning and fine tuning need the model to be pretrained on a large portion of the dataset. Currently, the widely used datasets from natural images, such as ImageNet [23] or Coco [24]. However, by considering the difference between SAR images and natural images (e.g., imaging mechanisms and target information [25,26]), features extracted from natural images via the pretrained model are not suitable for SAR images. Thus, it is imperative to use the SAR images to modify the weights of the pretrained model. Transfer learning treats the learned model as a feature extractor, as it changes the last few layers and only trains the parameters in the modified layers [27]. Unlike transfer learning, fine tuning takes the weights of trained models for initialization, modifies the top layers, and then trains the model with the target dataset. Therefore, based on the above analysis, fine tuning is adapted to train the ship classification model.

In addition, with deeper and increased deep-learning models, the features from the pretrained model become more abstract [28]. Since SAR images are different from natural images (e.g., range compression [26]), the greater features of pretrained models may not suitable for SAR images. To relieve this, a relatively shallow convolutional network (i.e., VGG [29]) is selected as our original model. Especially, VGG16, one kind of VGG, is the winner in the ILSVRC-2014 competition and has 23 layers. Compared with other deep learning models, such as InceptionV3 (159 layers), VGG19 (29 layers), and Xception (126 layers), VGG16 has a relatively shallow depth. It is used to construct a ship classification model in this paper. To evaluate the effectiveness of the proposed method, deeper models, such as VGG19 [29], are also used to evaluate our proposed method.

Based on the above analysis and to better exploit the benefits of deep learning with the capability to automatically learning features of structurally complex objects, VGG16 is used to construct a ship classification model in this paper. In addition, 4 models, including VGG16, VGG19, Xception [30], and InceptionV3 [31], and two training methods, including transfer learning and fine tuning, are conducted individually. The aim of this paper focuses on the application of convolutional neural networks (CNNs) to ship classification in high-resolution SAR images using small dataset. Compared to the feature-based method, CNNs automatically learn the discriminative features without human interference. To address the limited dataset when training the model, fine tuning is proposed to train the model with the consideration of SAR characteristics.

The organization of this paper is organized as follows. Section 2 relates the materials to the background of the VGG16 and our proposed ship classification model and also presents the workflow of our experiments, especially our training scheme with small datasets. Section 3 introduces our experimental results and analysis. Section 4 and Section 5 are the discussion and conclusions of this paper, respectively.

## 2. Materials and Method

### 2.1. Background on VGG16

The building blocks for VGG16 are a convolutional operation, pooling operation and an activation function, including rectified linear units (ReLu) and softmax. The convolutional operation is defined in Equation (1).

(1) $$\;z_{i_{0}j_{0}\;}=\sum_{i=1}^{m}\sum_{j=1}^{n}W_{ijc}X_{i_{0}+i,\;\;j_{0}+j}$$

where the size of the kernel is represented by m×n. X represents the input, and W represents the weights of the kernel in the cth channel.

For the pooling operation, there are two widely used pooling operations (i.e., maximum and average pooling), and they are expressed by Equations (2) and (3), respectively.

(2) $$\;y=\underset{1\leq i\leq m,\;1\leq j\leq n}{max\;}z_{i_{0}+i,j_{0}+j}$$

(3) $$\;y=\frac{\sum_{i=1\;}^{m}\sum_{j=1}^{n}z_{i_{0}+i,j_{0}+j}}{m\times n}$$

Even though there are many variations of activation functions (e.g., rectified linear units (ReLu), softmax, Tanh, and leaky ReLU), the most commonly used variations are ReLu and leaky ReLu in the feature extraction phase and softmax in the fully connected layers. These variations are expressed by Equations (4)–(6), respectively, and the others are referred to elsewhere [22]. α in Equation (5) is a hyperparameter [22]. ReLu is adapted in the original VGG16.

(4)  y=max(0, x )

(5)  y=max(αx, x )

(6) $$\;y_{i}=exp\left(X_{i}\;\right)/\sum_{j=1}^{C}X_{j}$$

**Figure 1:**
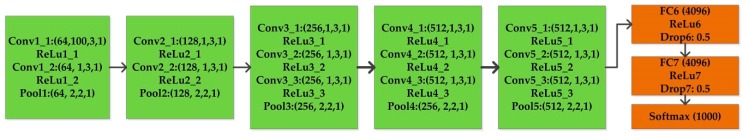
The architecture of VGG16. The green boxes indicate the convolutional blocks, and the brown boxes represent the fully connected blocks.

There are two architectures of VGG: VGG16 and VGG19. The main difference between them is that VGG19 has 3 more convolutional layers than VGG16. Therefore, compared with VGG19, VGG16 has a relatively shallow depth of neural networks. The building blocks of VGG16 can be divided into two groups: convolutional building blocks and fully connected (FC) layer blocks, as indicated by the green and brown rectangles in Figure 1, respectively. Convolutional building blocks distill image features that range from low-level to high-level. They consist of five convolutional layer groups (each layer is stacked with convolutional layers (Conv), rectified linear units (ReLu), and pooling layers (Pool)). Specifically, there are two Conv layers, two ReLu layers, and one Pool layer in the first two groups and there are three Conv layers, three ReLu layers, and one Pool layer for the last three groups. Each Conv layer has a kernel size of 3, the stride of 1, and the padding of 1 and generates an output feature map with units of 64, 128, 256, 512, and 512. For each Pool layer, the stride and kernel size are both 2. The four numbers that occur after each layer indicate the output number of the feature maps, padding, kernel size, and stride. Fully connected layers act as classifiers. The outputs of these three fully connected layers are 4096, 4096, and 1000, where 4096 is the number of hidden neural units in the fully connected layers and 1000 is the number of classes. Drop layers (Drop) are also used to avoid overfitting. The parameter after Drop indicates the random removal of neurons with a probability of 0.5.

### 2.2. Proposed Method

**Figure 2:**
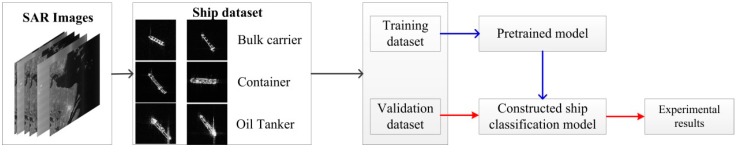
Experimental workflow. The black, blue, and red arrows represent the data processing, training, and test phases, respectively.

The experimental workflow is shown in Figure 2. Black, blue, and red arrows represent the phase of data processing, training, and testing, respectively. First, three categories of ship chips which include Bulk Carrier, Container, and Oil tanker, are obtained from the high-resolution COSMO-SkyMed Images. Then, these ship chips are split into training and validation dataset, with percentages of 70% and 30%, respectively. After that, the ship classification model based on VGG16 is constructed and trained on the basis of the pretrained VGG16 via the ImageNet dataset. Finally, the validation dataset is used to evaluate the performance of the trained model. The dataset is discussed later in Section 3.1, and the constructed ship classification and training strategies are the focus in this section.

#### 2.2.1. Ship Classification Model

**Figure 3:**
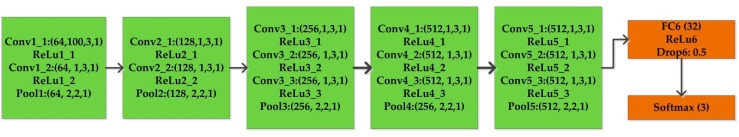
The architecture of the designed ship classification model based on VGG16.

Our proposed ship classification model is based on VGG16. Unlike the ImageNet and Coco dataset, there are only three categories. To better suit the SAR ship classification, the fully connected layer blocks are substituted with two fully connected blocks, as shown in Figure 3. The settings for the convolutional layers blocks are the same as those for VGG16. The main difference from VGG16 is the top layers. There are two fully connected layers with 4096 hidden units in VGG16, whereas there is only one layer with 32 hidden units in the ship classification model. For fully connected layer blocks, the dropout value is 0.5, and the output for softmax is 3. Since the training dataset is small, the number of neural units is the hyperparameter and will be discussed later in Section 3.2.1.

#### 2.2.2. Fine Tuning Ship Classification Model

There are two efficient ways to train deep-learning models with small datasets: fine tuning and transfer learning. These two methods first train the model on large public datasets, such as ImageNet, and then usually modify the top layers to accommodate a specific application. They have the advantages of training a deep learning model with small datasets. Besides, as stated in Ref. [32], the low level neural layers learned by deep learning models are similar to Gabor filters [32], which is useful for extracting features such as corners, edges [33]. So, we make the assumption that the lower level neural layers share common features. Here, the top layer is typically a fully connected layer. Both methods take the weights of the models in the low-level features as the inputs instead of random weights. The difference between them is the training process. Transfer learning only trains the newly modified features [32], whereas fine tuning not only trains the new layers but also the pretrained layers.

**Figure 4:**
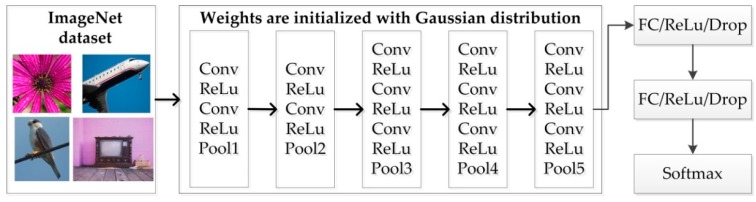
Workflow of training on the ImageNet dataset for VGG16.

**Figure 5:**
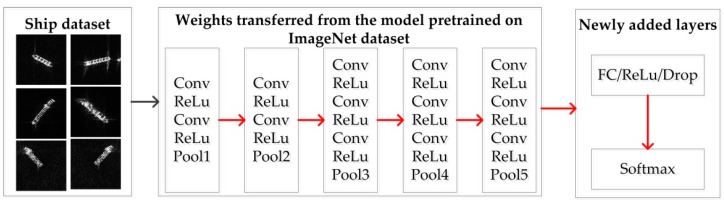
Workflow of training using fine tuning on the ship dataset for ship classification based on VGG16. The red arrows indicate the weights involving these layers will be learned.

**Figure 6:**
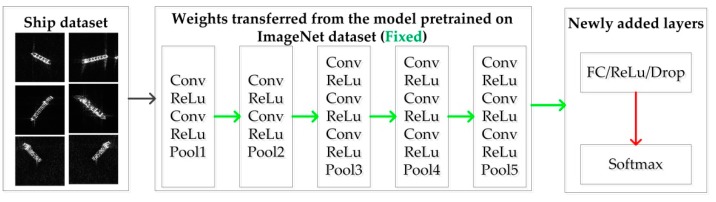
Workflow of training using transfer learning on the ship dataset for ship classification based on VGG16. The red arrows indicate that the weights involved in these layers will be learned, and the green arrows represent that the parameters will remain unchanged.

To better illustrate the difference between them, VGG16 is used as an example. First, VGG16 is trained using a public ImageNet dataset with weights initialized with the Gaussian distribution, as shown in Figure 4. During training, the weights (except for the top layers (i.e., fully connected layers)) are transferred to the model for ship classification, as shown in Figure 5 and Figure 6. It is obvious that the parameters for both fine tuning and transfer learning are initialized by those trained on the ImageNet dataset. Transfer learning maintains the transferred weights indicated by the green arrows and only learns the parameters of the newly added layers indicated by the red arrows, as shown in Figure 6, whereas fine tuning learns both parts (as indicated by the red arrows shown in Figure 5). Even transfer learning and fine tuning have the advantages of flexibility and robustness and are widely used in the natural image community [22] and remote sensing [19,20,33], but the weights of the low-level and mid-level convolutional blocks for transfer learning are fixed; thus, it may not suitable for SAR images. Therefore, fine tuning is utilized in this paper.

#### 2.2.3. Training Details

Our experiment was performed with a free and open operating system with the version 14.04 released by international Canonical Ltd, and an 8G memory NVIDAI GPU GTX1070. Our implementations are on the top of Keras [34], which is a deep-learning library that is built on the basis of TensorFlow, CNKT, and Theano. The pretrained models are downloaded from the Keras website. As stated in Section 2.2.2, transfer learning is also used to train the models to compare it with the performance of fine tuning. Fine tuning is learned by a stochastic gradient descent [22,35,36], with a learning rate of 0.0001 and a moment of 0.99. Transfer learning is guided by RMSprop [22,37], with a learning rate of 0.001 and a moment of 0.9. The conditions for termination are that the classification accuracy or loss almost remains the same.

## 3. Results

### 3.1. Experimental Data

#### 3.1.1. SAR Images

**Figure 7:**
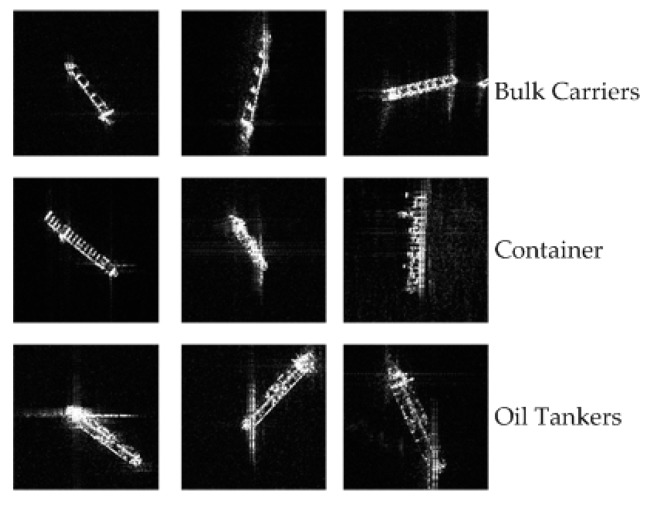
Examples of ship chips.

A total of 6 scenes from the COSMO-SkyMed level 1A HIMAGE (single-look complex Slant (SCS) product) images in the X-band with single HH or VV polarization are used to evaluate our approach, and the detailed information is shown in Table 1. The data are calibrated and converted into grey in the range [0, 255]. The ships are found automatically or manually on the SAR images. Three kinds of ships are obtained from these images through SAR experts and field experiments, and there are 146 bulk carriers, 156 containers, and 144 oil tankers used for classification. Some ship chips are shown in Figure 7. The bulk carriers have repeating textures due to the box-like hatches in the longitudinal direction. The containers also have repeating characteristics due to truck-size containers. The oil tankers have symmetric features because of the intense backscattering of pipelines along the centerline [3].

#### 3.1.2. Pretrained Models

To evaluate the effectiveness of our proposed method, three other models, including VGG19, Xception, and InceptionV3, are also used to construct our ship classification in these experiments. These four models can be divided into groups: the first two stem from VGG and the last two models evolve from GoogleNet [38]. The aim of GoogleNet is focused on the fact that the deeper the neural network is, the higher the performance. In addition, the inception module is used to increase both the width and depth of the neural network. There are five variations of GoogleNet, including GoogleNet, InceptionV2, InceptionV3, InceptionV4, and Xception. Considering the fact that InceptionV3 and Xception have the top performances regarding classification and are available now, these two neural architectures are used in this paper. InceptionV2 adds batch normalization to GoogleNet, and InceptionV3 exploits factorization ideas in the Inception and Xception variations, which introduces the depth-wise separable convolution of InceptionV3 [30]. These four models are pretrained on the ImageNet dataset, and the ship classification model based on them is constructed similarly to that based on VGG16, as shown in Section 2.2.1. To save energy when training these models on the ImageNet dataset, pretrained models are downloaded from the Keras website, and the information is shown in Table 2. It is obvious that VGG16 has a relatively shallow depth, which is equal to the sum of the input layer, Conv, Pool, FC, and an output layer.

### 3.2. Experimental Results and Analysis

#### 3.2.1. Influence of Units in the Fully Connected Layers

Considering that the number of neural units in the fully connected layers may have a potential impact on the classification results, comparison experiments are conducted with a different number of units in the fully connected layers 32, 64, 128, 256, and 4096. The classification accuracy and validation accuracy are shown in Table 3. In addition, precision, recall, and F_1_ score are also used to evaluate the influence of units in the fully connected layers. It is obvious that the number of units has little impact on the classification results from Table 3 with regard to the five metrics (e.g., training accuracy, validation accuracy, and F_1_ score) when using fine tuning to train the ship classification model. To evaluate the performance of these five models, McNemar’s test is used [39,40]. There is no significant difference among these models because the *p*-value is larger than the significance level of 0.05. Since the model has the minimum hidden neural units (32), which means few weights to be learned to avoid overfitting, it is used for our experiments.

#### 3.2.2. Comparison of Fine Tuning and Transfer Learning

**Figure 8:**
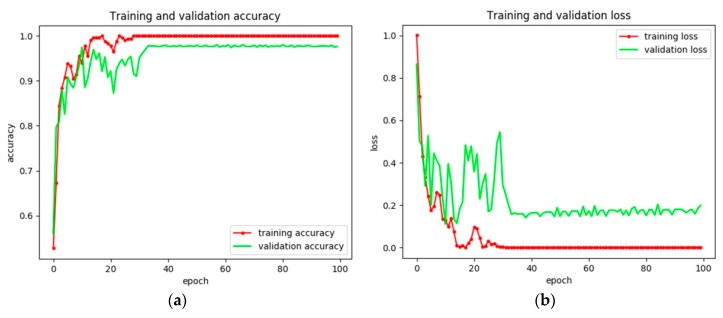
Experimental results of the ship classification model based on VGG16 via transfer learning. (**a**) Training and validation accuracy and (**b**) training and validation loss.

**Figure 9:**
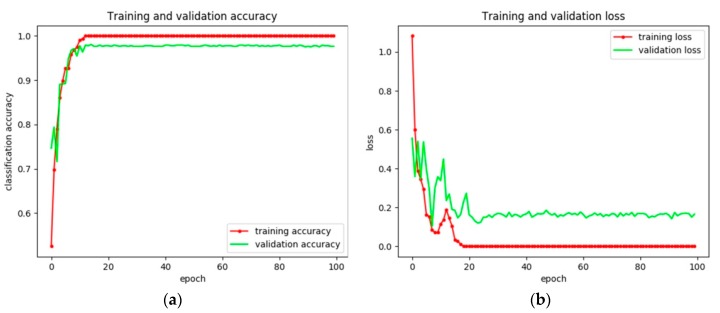
Experimental results of the ship classification model based on VGG16 via fine tuning. (**a**) Training and validation accuracy and (**b**) training and validation loss.

To evaluate the effectiveness of our proposed training method, the constructed ship classification model based on VGG16 is trained via fine tuning and transfer learning separately. The training and validation accuracies are shown in Table 4 and Figure 8 and Figure 9. On the one hand, it is obvious that fine tuning is more stable than transfer learning during the training process. On the other hand, even when the training accuracies of both methods are the same (i.e., 100%), the validation accuracy of the model trained by fine tuning is approximately 2% higher than that trained by transfer learning. The reason for this phenomenon may be that features in the deeper models are suitable for natural images and not SAR images. Besides, the time for training by transfer learning is less than that by fine tuning. This is because compared with transfer learning, fine tuning also modifies the weights in the transferred weights, which may cost time. For better clarification, the confusion matrix of the two training methods is shown in Table 5 and the precision, recall, and F_1_ score of each category are shown in Table 6.

McNemar’s test is also used and the contingency table is shown in Table 7. It is easy to obtain the fact that there is no significant difference between these two models. However, compared with the model trained via fine tuning, even the model via transfer learning can exactly classify oil tankers, but it failed to recognize the containers as shown in Table 5. Therefore, the precision, recall, and F_1_ score of the model trained by fine tuning are higher than those trained by transfer learning. In addition, it is obvious that F_1_ score via fine tuning is 2% greater that via transfer learning. The reason for this is that the features of the pretrained model may be suitable for natural images, not SAR images. Unlike transfer learning, fine tuning also modifies the weights of the low layers in the pretrained models, thus the features learned may suitable for SAR images. Since the weights of convolutional neural networks are fixed for the ship classification model, it may be beneficial for transfer learning in SAR images if the dataset of the pretrained models is from SAR images.

#### 3.2.3. Comparison with Other Models

**Figure 10:**
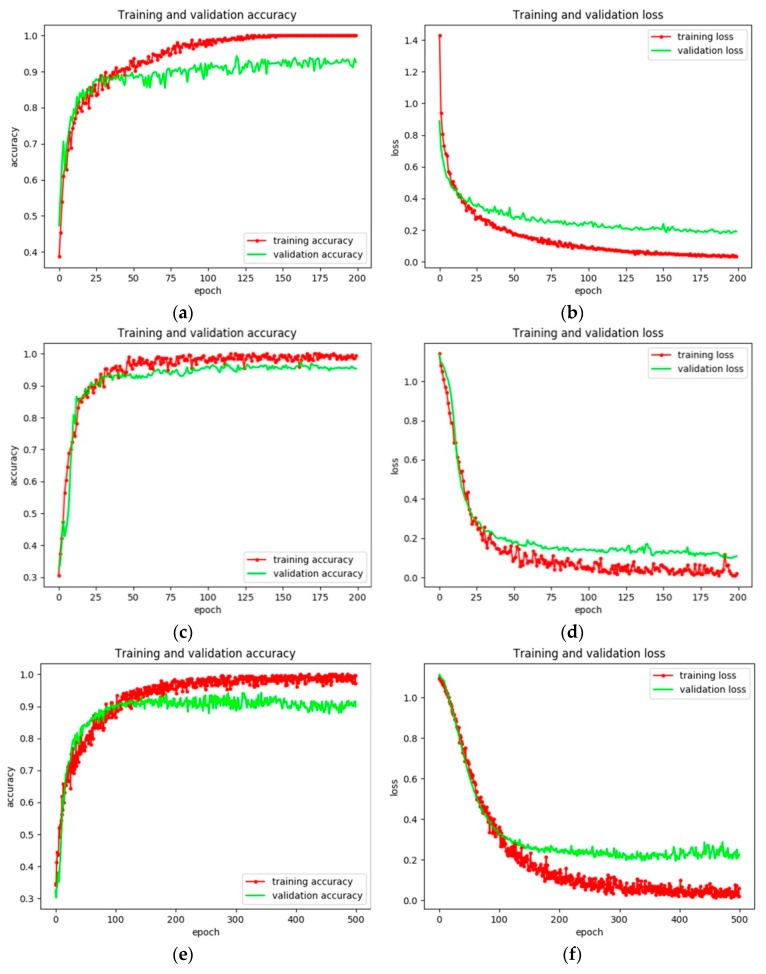
Training and validation accuracies of the ship classification model based on Xception. (**a**,**c**,**e**) show the training and validation accuracies, and (**b**,**d**,**f**) show the corresponding training and validation losses via the VGG19, InceptionV3, and Xception models, respectively.

Three other models are trained via fine tuning based on the same condition (i.e., the fully connected layers have one layer with 32 units). The training and validation accuracies are shown in Table 8 and Figure 10. McNemar’s test is also used. Except for the ship classification model based on Xception, the other two models are not significantly changed through through McNemar’s test. However, the VGG16 performs better than the other three models with regard to F_1_ score. It is apparent that VGG16 has the lowest Top-1 and Top-5 accuracies and the shallowest depths among these four models in the pretrained models from Table 2. However, the ship classification accuracy of VGG16 achieves the highest accuracies (at least 2% for the F_1_ score). This may be due to the fact that because of the differences between SAR images and natural images, features in the higher layers of the deeper model pretrained on the ImageNet dataset are suitable for natural images and not SAR images. To further evaluate the performance of these four models, the confusion matrix, precision, recall, and F_1_ score are used. The confusion matrices of these four models are shown in Table 5 and Table 9, and the metrics are shown in Table 10. It is obvious that the ship classification model based on VGG16 has the highest F_1_ score. In addition, it is easy to see that the higher classification on the ImageNet dataset is not guaranteed to be higher in the SAR images. For the first group, VGG16 with a relatively shallow depth has a 5% higher accuracy compared to VGG19; for the second group, InceptionV3 improves by almost 5% with regard to F_1_ score. The reason for this may be that transfer learning only trains the newly modified features, whereas fine tuning not only trains the new layers but also the pretrained layers. Thus, through training the model by fine tuning, the features may be more suitable for SAR images. Besides, compared with the other three models, the time of our proposed model has a fast speed. There are two reasons for this, i.e., the depth of the constructed model and the training times of these models. Since both the depth of VGG16 and the training time are the minimum, VGG16 has the fast training speed.

**Figure 11:**
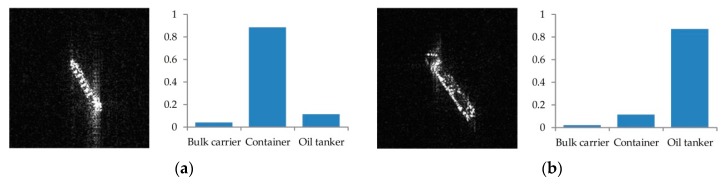
Examples of misclassified ships. (**a**) Bulk Carrier is classified as Container; (**b**) Container is misclassified as Oil Tanker. Their corresponding classification probabilities into three categories are also shown as indicated by royal blue histograms.

In addition, it is easy to note that bulk carriers and containers are misclassified as other kinds of ships in these four models. Examples of misclassified bulk carriers and containers are shown in Figure 11.

## 4. Discussion

Since the volume of ship constructed datasets is small, cross validation is important to evaluate the performance of the classifier. In this paper, 5-cross validation is used. Table 11 is the cross validation accuracy for transfer learning and fine tuning of VGG16 and fine tuning of VGG19. It is obvious that compared with transfer learning, fine tuning achieves higher average classification accuracy and lower standard deviation. This is because fine tuning also modifies the transferred weights, which may make the learned features suitable for SAR images.

Through the above experiments and analysis, it can be clearly seen that among the above four models, each model has its own advantages. The model based on VGG16 achieves the best F_1_ score and the best observations of Oil Tankers. The model based on VGG19 has the best observations of Bulk Carriers. The model based on InceptionV3 has the best precision for Oil Tankers and the best observations of Containers. The model based on Xception has the best precision for containers and the best observations of oil tankers. It will be our future goal to adapt ensemble learning [41] to take advantage of these models and achieve the best performance. However, among these four models, i.e., VGG16, VGG19, Xception, and InceptionV3, but not Xception, the other three models have no significant change for ship classification with McNemar’s test. This may be characteristic of this dataset and more ship classification datasets are needed to verify this in the future. In addition, due to the differences between SAR images and optical images, large SAR image datasets will be constructed to better exploit the methodology of deep learning, and future research will be conducted on pretrained models with SAR images, which may show the benefits of transfer learning and combined SAR inherent characteristics (e.g., statistical distributions with convolutional neural networks) to enhance the classification results of ship classifications. Not only does fine tuning achieve promising results, it also results in overfitting of the present model. The increase in datasets is likely to solve this problem.

## 5. Conclusions

In this paper, convolutional neural networks are used to classify ships in high-resolution SAR images with small datasets. Specifically, firstly, a SAR ship dataset is constructed from 6 COSMO-SkyMed images and, considering the characteristics of SAR images, a ship classification model based on VGG16 is constructed. Second, fine tuning is utilized to train the model, which is pretrained on an ImageNet dataset. The experimental results reveal that (1) our proposed ship classification model achieves the best performance with regard to classification accuracy, even with 5-cross validation; (2) compared with the model trained via transfer learning, the model trained by fine tuning has the best performance in classification accuracy; (3) compared with other models, the ship classification model based on VGG16 achieves at least 2% higher accuracies for classification. The inherent characteristics of SAR images will be combined with convolutional neural networks to enhance performance in future works. In addition, the ensemble of different deep-learning models will be used to improve performance.

## Figures and Tables

**Table 1:** Experimental SAR images information.

Sensors	Imaging Time	Pixel Spacing (Azi × Rg, m)	Near Range Incidence (°)	Band	Orbit
COSMO-SkyMed	12 July 2010	1.90 × 2.23	58.79	X	Descending
COSMO-SkyMed	12 July 2010	0.93 × 2.22	24.87	X	Descending
COSMO-SkyMed	12 July 2010	1.67 × 2.05	48.63	X	Ascending
COSMO-SkyMed	13 July 2010	1.90 × 2.23	58.79	X	Descending
COSMO-SkyMed	13 July 2010	1.66 × 2.05	48.63	X	Ascending
COSMO-SkyMed	14 July 2010	1.14 × 1.92	30.71	X	Descending

**Table 2:** Pretrained model information via the ImageNet dataset. During the inference for an image in ImageNet, the trained deep learning model outputs the probabilities of each class. The Top-1 accuracy means if the label corresponding to the maximum probability is correct, then this image is correctly classified. The Top-5 accuracy means if the label is included in the top-5 maximum probability, then this image is regarded as correctly classified.

Model	Top-1 Accuracy (%)	Top-5 Accuracy (%)	Depth
InceptionV3	78.8	94.4	159
VGG16	71.5	90.1	23
VGG19	72.7	91.0	26
Xception	79.0	94.5	126

**Table 3:** Experimental results with different units in the fully connected layers of the ship classification model based on VGG16.

The Number of Units	Training Accuracy (%)	Validation Accuracy (%)	Average Precision (%)	Average Recall	Average F_1_ Score
32	100	97.66	97.85	0.9774	0.9779
64	100	97.79	97.81	0.9774	0.9778
128	100	96.09	96.24	0.9632	0.9628
256	100	97.92	97.81	0.9774	0.9778
4096	100	97.01	97.00	0.9710	0.9705

**Table 4:** Classification accuracy of the ship classification model via fine tuning and transfer leaning.

Training Methods	Training Accuracy (%)	Validation Accuracy (%)	Training Time (s)
Fine tuning	100	97.66	811
Transfer learning	100	95.83	710

**Table 5:** Confusion Matrix of the model trained via transfer leaning and fine tuning on validation dataset.

	Transfer Learning			Fine Tuning		
	Bulk Carrier	Container	Oil Tanker	Bulk Carrier	Container	Oil Tanker
Bulk Carrier	41	1	1	41	2	0
Container	2	43	2	0	46	1
Oil Tanker	0	0	43	0	0	43

**Table 6:** Precision, recall, and F_1_ score of the model trained via transfer leaning and fine tuning on validation dataset.

Label	Transfer Learning			Fine Tuning		
	Precision	Recall	F_1_ Score	Precision	Recall	F_1_ Score
Bulk Carrier	0.9535	0.9535	0.9535	1.0	0.9535	0.9762
Container	0.9773	0.9149	0.9451	0.9583	0.9787	0.9684
Oil Tanker	0.9348	1.0	0.9663	0.9773	1.0	0.9885
Average total	0.9552	0.9561	0.9556	0.9785	0.9774	0.9780

**Table 7:** Contingency table of the models trained by fine tuning and transfer learning.

Model Trained by Transfer Learning	Model Trained by Fine Tuning	
	No. of Cor_cla	No. of Incor_cla
No. of cor_cla *	124	2
No. of incor_cla *	6	1

* No. of cor_cla and No. of incor_cla represent the number of correct and incorrect classification ships in the test dataset.

**Table 8:** Classification accuracies and training time of the four models.

Model Type	Training Accuracy (%)	Validation Accuracy (%)	Training Time (s)
VGG16	100	97.66	811
VGG19	100	92.48	1829
InceptionV3	99.37	95.48	2400
Xception	100	89.47	5500

**Table 9:** Confusion matrix of the model based on VGG19, InceptionV3, and Xception.

Model	VGG19			InceptionV3			Xception		
	Bulk Carrier	Container	Oil Tanker	Bulk Carrier	Container	Oil Tanker	Bulk Carrier	Container	Oil Tanker
Bulk Carrier	41	0	2	39	4	0	36	0	7
Container	4	41	2	0	47	0	5	40	2
Oil Tanker	1	1	41	1	1	41	0	0	43

**Table 10:** Precision, recall, and F_1_ score of the models based on VGG19, InceptionV3, and Xception.

Label	VGG19			InceptionV3			Xception		
	Precision	Recall	F_1_ Score	Precision	Recall	F_1_ Score	Precision	Recall	F_1_ Score
Bulk Carrier	0.8913	0.9535	0.9213	0.9750	0.9070	0.9397	0.8780	0.8372	0.8571
Container	0.9762	0.8723	0.9213	0.9038	1.0	0.9495	1.0	0.8511	0.9195
Oil Tanker	0.9111	0.9535	0.9318	1.0	0.9535	0.9762	0.8269	1.0	0.9053
Average total	0.9262	0.9264	0.9263	0.9596	0.9535	0.9565	0.9017	0.8961	0.8988

**Table 11:** Overall classification accuracies on the dataset for various network configurations using 5-fold cross validation.

Model	1	2	3	4	5	Mean	Standard Deviation
VGG16-FT *	98.11%	93.56%	100%	93.98%	92.42%	0.9561	0.0326
VGG16-TF *	93.98%	90.13%	100%	92.80%	91.23%	0.9363	0.0385
VGG19-FT *	91.29%	90.56%	93.13%	89.47%	92.05%	0.9130	0.0140

* VGG16-FT and VGG16-TF mean the ship classification model based on VGG16 are trained by fine tuning and transfer learning respectively. VGG19-FT means the ship classification model based on VGG19 trained by fine tuning.
